# Genotype and phenotype analysis of a cohort of patients with congenital hyperinsulinism based on DOPA-PET CT scanning

**DOI:** 10.1007/s00431-019-03408-6

**Published:** 2019-06-19

**Authors:** Jinwen Ni, Jingjie Ge, Miaoying Zhang, Khalid Hussain, Yihui Guan, Ruoqian Cheng, Li Xi, Zhangqian Zheng, Shuhua Ren, Feihong Luo

**Affiliations:** 1 Department of Endocrinology and Inborn Metabolic Diseases, Children’s Hospital of Fudan University, 399 Wanyuan Road, Shanghai, 201102 China; 2 PET CT Center, Division of Nuclear Medicine, Huashan Hospital, Fudan University, 518 East Wuzhong Road, Shanghai, 200235 China; 3 Department of Pediatrics, Division of Endocrinology, Sidra Medicine OPC, C6-340 PO Box 26999, Al Luqta Street Education City North Campus, Doha, Qatar

**Keywords:** Congenital hyperinsulinism, Hypoglycemia, Hyperinsulinemia, Mutation, Genetic association studies

## Abstract

Congenital hyperinsulinism (CHI) is a clinically, genetically, and morphologically heterogeneous disorder. ^18^F DOPA-PET CT scanning greatly improves its clinical outcome. Here, we presented the first Chinese ^18^F DOPA-PET CT scanning–based CHI cohort highlighting the variable ethic clinical phenotypes and genotypes. Fifty CHI patients were recruited. Median age at presentation was 2 days. Median fasting time was 2 h. Mean insulin level was 25.6 μIU/ml. Fifty-two percent of patients were diazoxide-unresponsive with significantly shorter fasting tolerance time and higher serum insulin level compared with the responsive patients. Seventy-four percent of patients experienced at least one adverse drug reaction. Tremendously increased focal lesions (32%) were detected and 75% of them were cured through surgery. Thirty-one nucleotide sequence changes were identified in 48% patients. Four novel variants (Q608X, Q1347X, Q289X, F1489S) in *ABCC8* gene and 2 novel variants (G132A, V138E) in *KCNJ11* gene were detected. Of the variants, 87.1% harbored in *ABCC* and *KCNJ11* genes. T1042Qfs*75 in *ABCC8* gene was the most common mutation.

*Conclusion*: Highly increased portion of focal lesion was presented in Chinese CHI patients compared with that of the previous reports. Intolerance to diazoxide was much more evident in Chinese or East Asian than other populations. Certain hotspot mutations harbored in Chinese CHI patients.

**Table:** 

**What is Known:** • ^*18*^*F DOPA-PET CT scanning can provide informative guidance for surgical procedure when medical therapy is not well responded in CHI patients*.	
**What is New:** • *Intolerance to diazoxide is much more evident in Chinese and East Asian CHI patients compared with the other ethnic populations*. • Novel mutations were detected in ABCC8 and KCNJ11 gene. Hotspot mutations such as T1042Qfs*75, I1511K, E501K, G111R in ABCC8 gene, and R34H in KCNJ11 gene are predominantly responsible for Chinese CHI patients.

**What is Known:**

• ^*18*^*F DOPA-PET CT scanning can provide informative guidance for surgical procedure when medical therapy is not well responded in CHI patients*.

**What is New:**

• *Intolerance to diazoxide is much more evident in Chinese and East Asian CHI patients compared with the other ethnic populations*.

• Novel mutations were detected in ABCC8 and KCNJ11 gene. Hotspot mutations such as T1042Qfs*75, I1511K, E501K, G111R in ABCC8 gene, and R34H in KCNJ11 gene are predominantly responsible for Chinese CHI patients.

## Introduction

Congenital hyperinsulinism (CHI) is the most common cause of persistent hypoglycemia in newborns and infants which usually presents with intractable severe hypoglycemia [11, 14]. An early diagnosis and appropriate management of CHI is mandatory to avoid subsequent brain damage and neurologic disabilities [11, 14]. Single or combined drugs’ therapy of CHI is the first choice; however, medically unresponsive is frequent. Adverse drug reaction could also lead to therapy suspension. Then the management of the disease was largely dependent on its pancreas histological lesion subtype [9].

There are two major histological subtypes of CHI, diffuse and focal forms, which are clinically identical but differ in histology, underlying genetic mechanisms and management regiments [2]. Diffuse form exhibits islet cell hyperplasia involving the entire pancreas while focal form represents a localized area of islet cell adenomatosis surrounded by an otherwise normal pancreas. However, both of the two forms are unable to be identified from each other using biochemical methods [11]. If medical therapy is unresponsive, CHI patients with diffuse form require a subtotal (80–94% removal of the gland) or near-total (95%) pancreatic resection to achieve euglycemia [5]. But patients with focal form can be cured completely through a limited pancreatic resection of the focal lesion [1]. Therefore, the pre-operative distinction between the diffuse and focal form is vitally important.

The routine imaging techniques, such as CT or MRI, have limited values in distinguishing the diffuse and focal forms of CHI. Pancreatic venous sampling or the pancreatic arterial calcium stimulation test in pediatric period is highly invasive, and not so accurate [24]. Genetic analysis of CHI-related genes could be informative, for example, paternal germline mutations in *ABCC8* or *KCNJ11* are associated with the focal form of CHI whereas biallelic recessive mutations are associated with the diffuse form [3, 11]. However, no CHI-related mutations can be found in about 50% of all cases worldwide [4, 15, 21, 23, 28]. Recently, an accurate and sensitive technique, positron emission tomography (PET) using fluorine-18-labeled L-dihydroxyphenylalanine (^18^F-DOPA), has been applied in CHI to help with pre-operative localization of the focal lesion, but mainly in Caucasian populations [12, 20, 22]. Recently, the local ^18^F-DOPA PET CT imaging successfully applied in the clinical settings [29]. So, in this study, we further explore the genotype-phenotype correlations and clinical prognosis in a cohort of Chinese CHI patients after ^18^F-DOPA PET CT scanning.

## Patients and methods

### Patients

Altogether, 50 Chinese CHI patients were admitted into the study. A controlled fasting test was done on the patients. The diagnosis of CHI was based on the following criteria: (1) consistently detectable insulin/C-peptide with blood glucose level < 2.6 mmol/l (46.8 mg/dL); (2) glucose infusion rate greater than 8 mg/kg per minute to maintain blood glucose greater than 3 mmol/l (54 mg/dL); (3) inappropriate hypoketosis and undetectable/low free fatty acids during hypoglycemia; (4) a positive glycemic response to glucagon. A 5-day trial of diazoxide (5–15 mg/kg/d in 3 divided doses) was started after diagnosis. The patients were defined as diazoxide unresponsive if intravenous dextrose could not be weaned or plasma glucose level of < 3.0 mmol/l during feeding and fasting periods. Diazoxide was discontinued in patients if there were obvious side effects such as gastrointestinal disturbances, edema, water-sodium retention, and congestive heart failure. So in these patients, the responsiveness to diazoxide was not completely known.

### ^18^F-DOPA-PET CT scan

The ^18^F-DOPA-PET CT scan was performed in all CHI patients at Huashan Hospital, Fudan University. Under general anesthesia, all the patients were fasted for at least 6 h, received intravenous dextrose infusion to control blood glucose level. Administration of glucagon was suspended 2 days before the procedure. A PET scan of the abdomen was done using a hybrid machine (Siemens BiographTM Truepoint PET/CT, Siemens Healthcare, Erlangen, Germany) 60 min after i.v. administration of ^18^F-DOPA (6 MBq/Kg). ^18^F-DOPA was produced by the nucleophilic method and was approved for clinical use in China by the National Medical Products Administration. A contrast-enhanced CT was also sequentially performed. Standard uptake value (SUV) was calculated between the area with the highest uptake and the rest of the pancreas. A SUV ratio > 1.5 was considered indicative of a focal lesion [13].

### Histology

Diazoxide-unresponsive patients and patients with focal type identified by ^18^F DOPA-PET CT scanning underwent pancreatectomy. The results of PET scan were made available to surgeons to help identify the potential lesion. During surgery, biopsies from the head, body, and tail of the pancreas were examined. Hematoxylin and eosin (H&E)-stained and immunohistochemical analysis was done to differentiate the histologic form of CHI. The determination of focal versus diffuse lesion was made on the permanent histologic sections by two pathologists who were masked to the results of the PET scan.

### Genetic analysis

Genomic DNA was extracted from peripheral leukocytes of the patients using QIAamp DNA Blood Mini Kit (Qiagen, Hilden, Germany). The protein-coding regions of human *ABCC8* (NM_000352.4), *KCNJ11* (NM_000525.3), *GCK* (NM_000162.3), *GLUD1* (NM_005271.3), *HADH* (NM_001184705.2), *HNF4A* (NM_000457.4), *SLC16A1* (NM_003051.3), and *UCP2* (NM_003355.2) genes was analyzed using Ion Torrent PGMTM [4]. The variants were validated by direct Sanger sequencing using an automated sequencer (ABI 3130 Genetic Analyzer; Applied Biosystems, Foster City, CA). Human Gene Mutation Database (http://www.hgmd.cf.ac.uk/ac/index.php), Online Mendelian Inheritance in Man Database (http://www.ncbi.nlm.nih.gov/omim), Single-Nucleotide Polymorphism Database (dbSNP 137, http://www.ncbi.nlm.nih.gov/snp), and 1000 Genomes Project (http://www.1000genomes.org/) were consulted to evaluate all the filtered variants. Furthermore, the Polymorphism Phenotyping (PolyPhen-2; http://genet-ics.bwh.harvard.edu/pph2), Sorting Intolerant From Tolerant (SIFT; http://sift.jcvi.org), and Mutation Taster (http://www.mutationtaster.org/) were used to test the pathogenicity of all the variants. ESEfinder (http://rulai.cshl.edu/tools/ESE/) was used to detect alterations in exonic splicing enhancers (ESE) due to nucleotide changes. Changes affecting highly conserved positions were generally interpreted as having damaging effects. Related variants were assessed in 50 normal controls.

### Statistical analysis

Data was analyzed using SPSS software version 17.0. Qualitative data are expressed in percentage. Quantitative data were expressed as mean ± standard deviation. Quantitative data consistent with the homogeneity of variance were analyzed using *t* test; otherwise, the Wilcoxon rank sum test was used. The statistical significance level was set at 0.05.

## Results

### Clinical background of the CHI patients

Among the 50 CHI patients (29 male and 21 female), no remarkable hypoglycemia or diabetes family history was found. The mean birth weight was 4007.6 ± 645.3 g for male and 3810 ± 589.7 g for female, both of which were above the 90th percentile of the national reference standard. The median age at presentation was 2 days after birth (range, 0 days to 11 years). At diagnosis, the median fasting time were 2 h (range, 0.3 to 12 h). The mean blood glucose was 2.1 mmol/l (range, 1.1 to 2.6 mmol/l), while the mean insulin level was 25.6 μIU/ml (range, 2.5–156 μIU/ml). To maintain blood glucose greater than 3 mmol/l (54 mg/dl), the mean required glucose infusion rate was 11.3 mg/kg·min (range, 8–20 mg/kg·min). Besides, 20 (40%) patients needed extra continuous glucagon intravenous infusion in a mean rate of 8.5 μg/kg·h (1.6–20 μg/kg·h) to achieve euglycemia. The serum ammonia was within normal range and β-hydroxybutyrate/non-esterified fatty acids were low in all patients.

### Clinical course

**Fig. 1:**
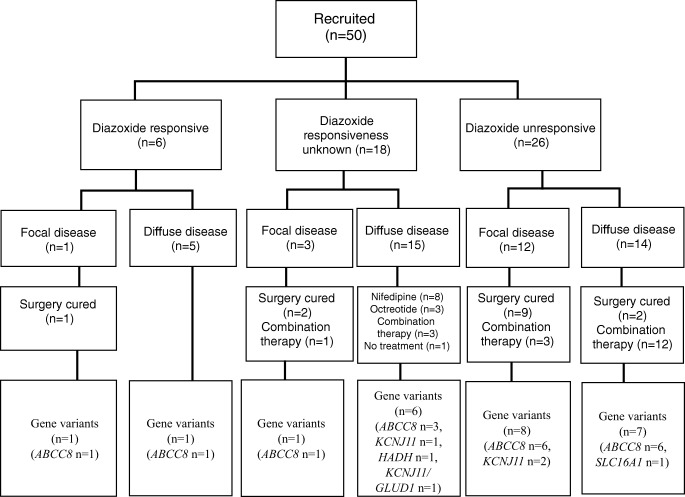
Summary of clinical course, ^18^F-DOPA-PET CT findings, and genotypes of 50 Chinese patients

After a 5-day trial of diazoxide, 6 (12%) patients were responsive while 26 (52%) patients were unresponsive. The mean fasting time was significantly lower in the unresponsive patients (2.5 ± 2.4 h) compared with that in the diazoxide-responsive patients (6 ± 3.3 h, *P* < 0.05). During hypoglycemia periods, the serum insulin level was significantly increased in diazoxide-unresponsive patients (mean insulin level, 29.6 ± 32.7 μIU/ml) compared with the diazoxide-responsive patients (mean insulin level, 11.5 ± 8.6 μIU/ml, *P* < 0.05). After given diazoxide, 37(74%) patients experienced at least one adverse drug reaction (ADR). Eighteen (36%) patients’ responsiveness to diazoxide was unknown due to side effect–related therapy suspension (Fig. 1). Gastrointestinal disturbances, circulatory complications, hypertrichosis was the most commonly observed in 48.4%, 41.9%, and 41.9% patients respectively.

### ^18^F-DOPA-PET CT scanning

^18^F-DOPA-PET CT scanning revealed that, in the 50 CHI patients, 16 (32%) patients had focal lesion while the other 34 (68%) had diffuse lesion. Among the 16 focal type patients, 1 patient (6.3%) was diazoxide responsive while 12 (75%) were unresponsive and three (18.7%) patients’ diazoxide-responsiveness was unknown. Similarly, in 34 patients with diffuse lesion, 14 (41.2%) were diazoxide-unresponsive while 5 (14.7%) were diazoxide-responsive and 15 (44.1%) patients’ diazoxide-responsiveness were unknown (see Fig. 1). Compared with the diffuse-type patients (median age, 13 days; range, after birth to 11 years), the focal-type patients were significantly younger at presentation (median age, 1.5 days; range, 6 h to 3 months) (*P* < 0.05). The mean fasting time (2.1 ± 1.5 h) of the focal-type patients were significantly shorter than that of the diffuse-type patients (4.4 ± 3.6 h) (*P* < 0.05).

### Clinical outcome with and without pancreatectomy

Totally, 14 patients (12 focal-type and 2 diffuse-type) received pancreatectomy. Among them, 2 patients with diffuse lesion and 1 patient with 2 focal lesions received near-total pancreatectomy (Table 1). Eleven patients with focal lesion underwent subtotal pancreatic resection. The resected tissue’s histological findings were consistent with the ^18^F-DOPA-PET CT scan results. Under normal feeding, no patient had hypoglycemia after the surgery. Among the 36 nonsurgically treated patients, except one patient with diffuse lesion who only need frequent feeding to achieve euglycemia, 5 patients were controlled with diazoxide, 3 patients using octreotide, 8 patients using nifedipine plus frequent feeding, and 19 patients were in combination therapy with diazoxide, octreotide, and nifedipine, including 4 patients with focal disease on PET CT as the proposal of pancreatectomy was rejected by their parents (Fig. 1).

**Table 1:** Summary of CHI patients who underwent pancreatectomy

Patient no.	PET CT	Pancreatectomy	Diazoxide responsiveness	Mutation
1	Focal	Partial (tail, 30%)	Unresponsive	–
2	Focal	Partial (tail, 30%)	Unresponsive	–
3	Focal	Partial (head, 25%)	Unresponsive	*ABCC8* c.4039C > T p.Q1347X
4	Focal	Partial (head, 20%)	Responsive	*ABCC8* c.331G > A p.G111R
5	Focal	Near-total (head and body, 90%)	Unresponsive	*KCNJ11* c.413 T > A p.V138E
6	Focal	Partial (head, 30%)	Responsiveness unknown	–
7	Focal	Partial (body, 50%)	Unresponsive	*ABCC8* c.2691delC p. W898Gfs*5
8	Focal	Partial (head, 30%)	Unresponsive	*KCNJ11* c.602G > C p.R201P
9	Focal	Partial (tail, 40%)	Unresponsive	–
10	Focal	Partial (body and tail, 60%)	Unresponsive	–
11	Focal	Partial (tail, 25%)	Responsiveness unknown	*ABCC8* c.742C > T p. R248X; c.2690A > T p.D897V
12	Focal	Partial (body, 50%)	Unresponsive	*ABCC8* c.3124_3126delins13 p.T1042Qfs*75
13	Diffuse	Near-total (90%)	Unresponsive	–
14	Diffuse	Near-total (95%)	Unresponsive	*ABCC8* c.4307G > A p.R1436Q; c.1887_1888delCAinsA

### Genetic analysis

A total of 31 nucleotide sequence changes within the CHI-related genes were identified in 24 of 50 (48%) patients. Most of which involved the *ABCC8* and *KCNJ11* genes (27/31, 87.1%) found in 22 of 50 (44%) patients. Among them, 18 patients (36%) harbored 23 *ABCC8* gene variants with 4 novel (Q289X, Q608X, Q1347X and F1489S) variants. Four patients (8%) harbored 4 *KCNJ11* variants with 2 novel missense variants (G132A, V138E). The other sequence changes were in *SLC16A1* (*n* = 1), *HADH* (*n* = 2), and *GLUD1* (*n* = 1). Fifteen of the 23 *ABCC8* gene variants and 4 *KCNJ11*, 2 *HADH* and the *GLUD1* variants found in this study were predicted to be diseasing causing by online mutation prediction tools (Table 2). The rest variants were predicted to be benign.

**Table 2:** CHI-related variants found in 50 Chinese patients

Mutation	PolyPhen-2	Mutation taster	Previous reported mutation	Age at presentation
ABCC8				
c.1822C > T p.Q608X	–	Disease Causing	Novel	1 h
c.4039C > T p.Q1347X	–	Disease Causing	Novel	15 h
c.4307G > A p.R1436Q	Probably damaging	Disease Causing	Y	30 min
c.1887_1888delCAinsA	–	Disease Causing	Y	30 min
c.1501G > A p.E501K	Probably damaging	Disease Causing	Y	11 days
c.331G > A p.G111R	Probably damaging	Disease Causing	Y	2 days
c.721del p.H241lfs;	–	Disease Causing	Y	3 days
c.563A > G p.N188S;	Possible damaging	Disease Causing	Y	3 days
c.2691delC p. W898Gfs*5	–	Disease Causing	Y	2 days
c.4466 T > C p.F1489S	Possible damaging	Disease Causing	Novel	1 day
c.742C > T p. R248X	–	Disease Causing	Y	1 day
c.2690A > T p.D897V	Probably damaging	Disease Causing	Y	1 day
c.865C > T p.Q289X	–	Disease Causing	Novel	5 months
c.3124_3126delins13 p.T1042Qfs*75	–	Disease Causing	Y	20 h
c.4532 T > C, p.I1511T	Possible damaging	Disease Causing	Y	2 h
KCNJ11				
c.101G > A p.R34H	Probably damaging	Disease Causing	Y	1 h
c.413 T > A p.V138E	Possible damaging	Disease Causing	Novel	1 day
c.602G > C p.R201P	Probably damaging	Disease Causing	Y	2 days
c.395G > C p.G132A	Probably damaging	Disease Causing	Novel	1 h
GLUD1				
c.1564A > G p.M522 V	Possible damaging	Disease Causing	Y	1 h
HADH				
c.29G > C p.R10P	Probably damaging	Disease Causing	Y	2 years and 2 months
c.89 T > A p.V30E	Probably damaging	Disease Causing	Y	2 years and 2 months

Compared with the rest with/without CHI-related mutation (mean age, 248.5 days), the patients with *ABCC8* or *KCNJ11* mutation were younger at presentation (mean age, 10.8 days for *ABCC8* and 0.8 days for *KCNJ11*) (*P* < 0.05). 9.5% patients with *ABCC8*/*KCNJ11* mutation were responsive to diazoxide. However, there was no statistical difference in blood glucose, serum insulin level, and glucose infusion rate between the patients with and without CHI-related mutation.

## Discussion

CHI is a clinically and genetically heterogeneous disorder with dysregulation of insulin secretion from pancreatic β-cells. As a national referral center, our patients come from 78.1% provinces of China. We found that only 12% CHI patients were diazoxide side effect–tolerant and responsive and 52.0% CHI patients were diazoxide-unresponsive, higher than previously reported of other Chinese patients [7, 26]. Compared with the others, the diazoxide-unresponsive patients showed shorter fasting tolerance time and higher serum insulin level.

Relatively high portion of our patients were intolerant to diazoxide and the therapy was halted in 36% patients. Gastrointestinal disturbances were the most common, occurred in 48.4% patients, such as poor appetite, nausea, and vomiting. About 6.5% patients relied on gastric tube feeding. Though diuretic courses were started with diazoxide in all patients, 41.9% patients had circulatory complications, such as high heart rate, edema, and fluid retention, especially in the first week after the initiation of diazoxide when intravenous dextrose was still needed. 12.9% patients had congestive heart failure when combined with infection. Hypertrichosis was noted in 22.6% patients especially 2–3 months after the diazoxide treatment. Similar situations were found in Japanese patients and Beijing cohort, China [6, 7]. After given diazoxide, 30.5% Japanese patients had adverse drug reaction, including hypertrichosis, edema, anemia, and cardiac failure [6]. However, only 14–18% persistent hypoglycemia patients were diagnosed with edema after given diazoxide in the USA [8, 10]. Moreover, other adverse drug reactions were less common in Asian patients. For example, neutropenia, thrombocytopenia and hyperuricemia found in 15.6%, 4.7%, and 5.0% patients in the USA [10], occurred in 1%, 0.8%, and 0.5% Japanese patients respectively [6]. All of these highly suggested different racial susceptibility to diazoxide in Chinese or East Asian populations. Careful surveillance for common adverse effects involving digestive and cardiovascular systems is warranted.

To date, variants in genes, such as *ABCC8*, *KCNJ11*, *GLUD1*, *GCK*, *HADH*, *SLC16A1*, *UCP2*, *HN4A*, and *HNF1A* are known to cause CHI. In our study, variants in CHI-related genes were found in 48% patients. Potential disease-causing mutations in *ABCC8/KCNJ11* gene were the most common, occurring in 44% CHI patients but much less than that in Japanese (61.3%) and Korean patients (82.0%) [21, 28]. It was noted that T1042Qfs*75, I1511K, E501K, and G111R in *ABCC8* gene and R34H in *KCNJ11* gene reoccurred in patients from different regions of China. Patients with the same mutation shared similar clinical characteristics, suggesting that potential hotspot mutations might existed in Chinese CHI patients. T1042Qfs*75 in *ABCC8* gene was found in two patient of this study and two patients in our hospital reported before [4]. The patients with T1042Qfs*75 mutation in *ABCC8* gene were diazoxide-unresponsive and had diffuse lesion in pancreas. I1511K in *ABCC8* gene was found in two patients of our study. E501K, G111R in *ABCC8* gene and R34H in *KCNJ11* gene was found in one of our patients and one reported previously [26]. Both the patients had I1511K mutation in *ABCC8* gene and the patients with R34H mutation in *KCNJ11* gene had diffuse lesion and were diazoxide-responsive. The patients with E501K mutation in *ABCC8* gene were diffuse-type and diazoxide-unresponsive. The patients with G111R mutation in *ABCC8* gene were diazoxide-responsive but had a focal lesion in pancreas. With the widespread adoption of genetic diagnosis in clinic, more mutational hotspots could be found, which would provide more detailed information to guide the management of CHI in Chinese patients.

**Fig. 2:**
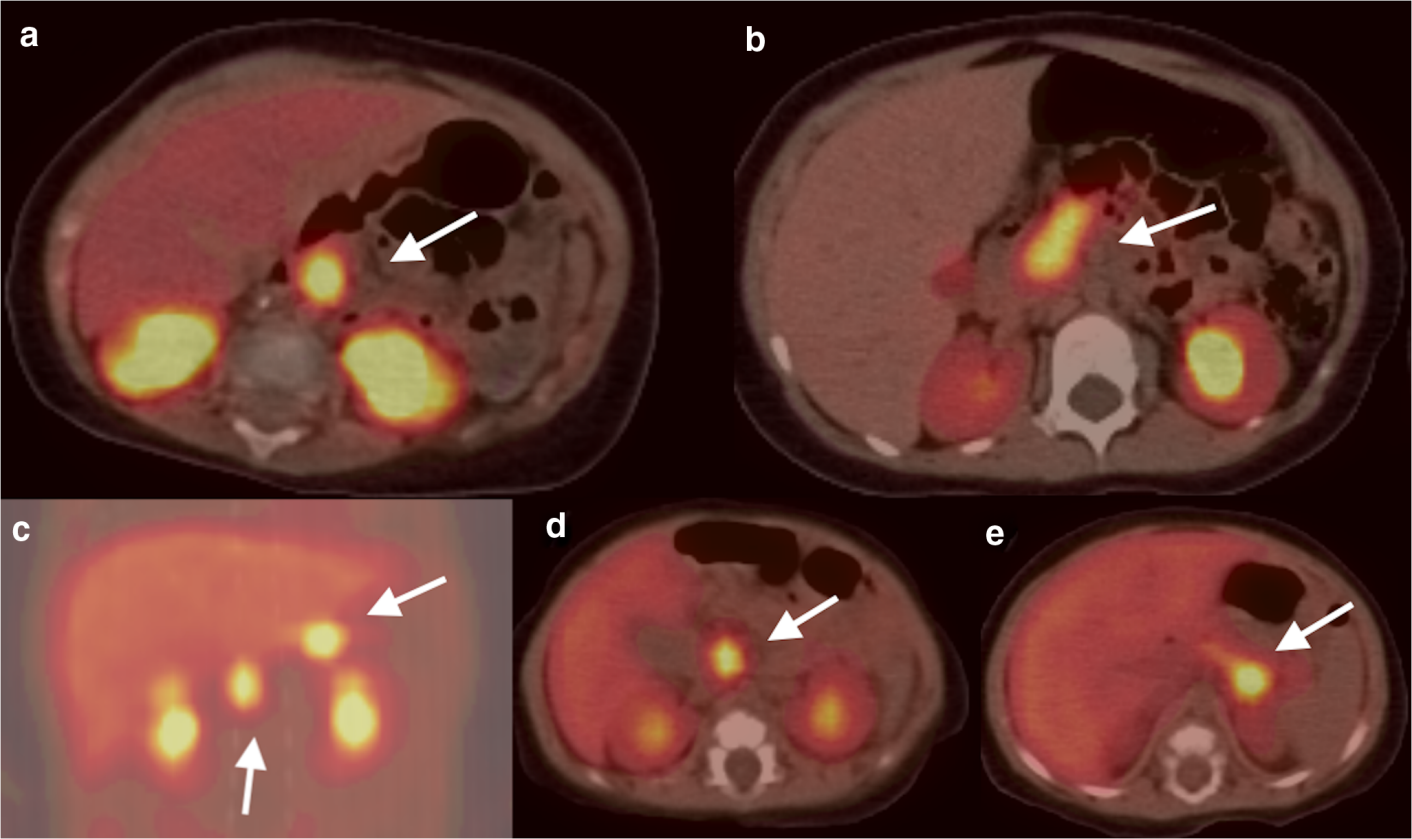
Typical ^18^F-DOPA-PET CT images in Chinese CHI patients. Focal lesion in the head of the pancreas (**a**, white arrow); diffuse lesion in the pancreas (**b**, white arrow); focal lesions both in the head and body of pancreas (**c**, **d**, **e**, case 5, KCNJ11 V138E mutation, white arrow)

In our study, we discovered 4 novel variants in *ABCC8* gene and 2 novel variants in *KCNJ11* gene. In the 4 novel variants in *ABCC8* gene, 3 were nonsense mutation as Q608X, Q1347X, and Q289X, which resulted in an incomplete and usually nonfunctional protein product. The other novel missense variant, F1489S in the *ABCC8* gene, was predicted as disease-causing. All four patients with the novel mutation were diazoxide-unresponsive. The patient with Q1347X mutation had a focal lesion and was surgically cured, while the other three patients were diffuse-type and were managed with combined therapy of diazoxide, nifedipine, and octreotide. The other two novel *KCNJ11* variants, G132A and V138E, were both predicted as disease-causing. The patient with G132A had a diffuse disease and was controlled by octreotide and nifedipine. However, V138E variant in *KCNJ11* gene caused two focal lesions (Fig. 2) in the patient who was cured through near-total pancreatectomy. Pathology results confirmed islet cell adenomatosis in the uncinate process and body of the patient’s pancreas.

Apart from the patients with novel mutation, the other patients with *ABCC8/KCNJ11* variants were mostly diazoxide-unresponsive and younger at presentation compared with other forms. 62.5% of focal-type patients in our study were found to have *ABCC8/KCNJ11* mutation. The findings in our study supported the notion that CHI patients with *ABCC8*/*KCNJ11* mutation were more difficult to manage through medical therapy but more likely to be cured through surgery after the historical type determination by ^18^F-DOPA-PET CT scan. ^18^F-DOPA-PET CT scan is highly sensitive and specific in helping with differentiating between diffuse and focal form of CHI [25, 27]. The complete excision of the lesion, especially the focal lesion, greatly relies on the preoperative ^18^F-DOPA-PET CT and the intraoperative histological confirmation. Without ^18^F-DOPA-PET CT scan, the outcome of surgical treatment is uncertain [7]. In all 50 CHI patients of our study, 16 (32%) patients had focal lesion while 34 (68%) had diffuse subtype, identified by ^18^F-DOPA-PET CT. After considering the result of ^18^F-DOPA-PET CT scan, 12 patients with focal lesion and 2 patients with diffuse lesion were cured through partial or near-total pancreatectomy (Table 1). The histopathological findings were consistent with the ^18^F-DOPA-PET CT scan results. Confirmed by histopathologic analysis, the focal lesion percentage of our study is 24% (12/50), lower than that in the North America report (50.5–52.8%) [16, 23] and that in the UK (27.8%) [19], but tremendously increased than that in the previous reports of Chinese surgical patients without ^18^F-DOPA-PET CT scan which was only around 5.3 to 7.7% [17, 18]. In combination with our previous report [29], the local ^18^F-DOPA-PET CT scan is comparable sensitive and specific to findings from other countries [25, 27] and demonstrates remarkable benefits in improving the management of CHI in China.

In summary, despite the comparable diazoxide-unresponsiveness in our patients to that in Caucasian CHI patients, the high intolerance to diazoxide is evident in Eastern Asian patients. Hotspot and sporadic novel *ABCC8* and *KCNJ11* gene mutations are predominantly responsible for Chinese CHI patients. A comprehensive etiological diagnosis using both genetic analysis and ^18^F-DOPA-PET CT scan will contribute to a better management and prognosis of CHI patients.

## Abbreviations

CHI: Congenital hyperinsulinism
^18^F-DOPA: Fluorine-18-labeled L-dihydroxyphenylalanine
PET: Positron emission tomography
ADR: Adverse drug reaction
